# High-Intensity Focused Ultrasound (HIFU) as salvage therapy for radio-recurrent prostate cancer: predictors of disease response

**DOI:** 10.1590/S1677-5538.IBJU.2017.0025

**Published:** 2018

**Authors:** Shawn Dason, Nathan C. Wong, Christopher B. Allard, Jen Hoogenes, William Orovan, Bobby Shayegan

**Affiliations:** 1 Division of Urology, McMaster University, Hamilton, ON, Canada

**Keywords:** High-Intensity Focused Ultrasound Ablation, Prostatic Neoplasms, Erectile Dysfunction

## Abstract

**Background:**

Some men with localized radio-recurrent prostate cancer may benefit from salvage high-intensity focused ultrasound (HIFU). Herein, we describe oncologic outcomes and predictors of disease response after salvage whole gland HIFU from our prospective cohort.

**Materials and Methods:**

Patients with localized radio-recurrent prostate cancer were prospectively enrolled from January 2005 to December 2014. Participants had to meet both biochemical and histological definitions of recurrence. Exclusion criteria included the receipt of prior salvage therapy, presence of metastatic disease, and administration of ADT in the 6-months prior to enrollment. Participants were treated with a single session of whole-gland HIFU ablation with the Ablatherm^TM^ device (EDAP, France). The primary endpoint was recurrence-free survival (RFS), defined as a composite endpoint of PSA progression (Phoenix criteria), receipt of any further salvage therapy, receipt of ADT, clinical progression, or death. Kaplan-Meier survival analysis was used to determine the primary end-point and stratifications were used to determine the significance of 6 pre-specified predictors of improved RFS (TRUS biopsy grade, number of study entry TRUS biopsy cores positive, palpable disease at study enrollment, pre-HIFU PSA, an undetectable post-HIFU PSA nadir, and receipt of prior hormone therapy). Survival analysis was performed on participants with a minimum of 1-year follow-up.

**Results:**

Twenty-four participants were eligible for study inclusion with a median follow-up of 31.0 months. Median PSA at study entry was 4.02ng/ml. Median time to PSA nadir was 3 months after treatment and median post-HIFU PSA nadir was 0.04ng/ml. Median 2-year and 5-year RFS was 66.3% and 51.6% respectively. Of our 6 prespecified predictors, an undetectable PSA nadir was the only significant predictor of improved RFS (HR 0.07, 95% CI 0.02-0.29, log-rank P<0.001). One participant underwent an intervention for a urethral stricture. No participants developed osteitis pubis or rectourethral fistulae.

**Conclusions:**

Salvage HIFU allows for disease control in selected patients with localized radio-recurrent prostate cancer. An undetectable PSA nadir serves as an early predictor of disease response.

## INTRODUCTION

Patients treated with primary radiotherapy for prostate cancer have a 20-60% risk of biochemical recurrence (1, 2). Various treatment options for biochemical failure are available, ranging from watchful waiting with delayed androgen deprivation therapy (ADT) to local salvage therapies. Over 90% of radio-recurrent patients receive ADT, which is not curative, associated with well-known side effects, and expensive (1). In one large series published by Zelefsky et al., a positive biopsy was present in 25% of patients receiving 81Gy dose of radiotherapy (3). Of the patients with a positive biopsy, 97% had a PSA relapse and 31% developed metastases within 10 years (3).

Although there is no widely accepted “gold standard” for salvage therapy for radio-recurrence of prostate cancer, some consider the gold standard for curative local salvage therapy as salvage radical prostatectomy (4). Salvage radical prostatectomy has been reported to have 5-year and 10-year biochemical recurrence free rates of 47-82% and 28-53% respectively (5). Cancer specific survival has been reported to range from 70-83% and 54-89% at 5-years and 10-years respectively (5). Despite these outcomes, salvage radical prostatectomy is rarely performed due to its high morbidity rate. In the radio-recur-rent setting the risk of intra-operative bowel injury is significantly higher and, at times, it is impossible to safely perform salvage radical prostatectomy (4). Other local therapies including cryotherapy (6) and brachytherapy (7) have also been used in smaller series as a salvage therapy for radio-recurrent prostate cancer, with a 50-70% biochemical recurrence free rate at 5 years.

High-intensity focused ultrasound (HIFU) is a minimally invasive local ablative technology currently being investigated in prostate cancer. Use of HIFU has been previously reported in the primary setting, as well as in the salvage setting for radio--recurrent prostate cancer (2, 8, 9). In the salvage setting, there are heterogeneous HIFU techniques, a lack of large prospective trials, and no consensus regarding the ideal candidate for salvage HIFU. To this end, we report our oncologic outcomes and predictors of disease response with whole-gland HIFU as salvage therapy for radio-recurrent prostate cancer.

## MATERIALS AND METHODS

Participants with radio-recurrent prostate cancer were prospectively enrolled in this institutional review board approved study from January 2005 to December 2014. Participants were offered inclusion in this study if they had experienced radiation failure after primary radiotherapy (external beam radiotherapy [EBRT] or brachytherapy [BT]) for prostate cancer. Prior to study enrollment, participants underwent clinical assessment, PSA testing, computed tomographic (CT) scan of the abdomen and pelvis, bone scan and transrectal ultrasound (TRUS) guided prostate biopsy. The TRUS guided biopsy template was intended to sample the entire gland, including a classic sextant biopsy and approximately 2 additional lateral cores on each side.

Study participants had to meet both biochemical and histopathologic definitions of failure. Biochemical failure was defined as either a PSA rise to 2ng/ml or more above their post radiotherapy PSA nadir (ASTRO Phoenix criteria (10)) or 3 consecutive PSA rises above their post radiotherapy PSA nadir (1997 ASTRO consensus criteria (11)). Participants for which a c bounce phenomenon was suspected due to an increase in years 2-3 post radiotherapy were followed for 6-18 months (12) to ensure that they were experiencing a true biochemical failure prior study enrollment. Participants also had to have histopathologic evidence of failure on a TRUS guided biopsy performed at study entry demonstrating prostate cancer. Participants were included in study analyses if they had a minimum of 1-year of follow-up. Study exclusion criteria included the receipt of prior salvage therapy or prior HIFU, the presence of metastases on staging investigations, and the receipt of ADT in the 6-months prior to biochemical determination of radiation failure.

Participants were treated with HIFU with the Ablatherm™ HIFU device (EDAP, France) using a standardized protocol similar to primary HIFU treatment. Treatment was done in a single session and monitored real-time with ultrasound to ensure whole-gland ablation. Spinal anesthesia with adjunctive intravenous sedation was used during treatment. Standardized device settings were employed - 100% acoustic power and 41-48 Watts of energy was used. The pulse duration was 6 seconds and there was a 4 second delay between each pulse. Pre-HIFU androgen deprivation therapy and transurethral resection of prostate were not utilized. Participants were sent home the same day with a urethral Foley catheter.

After the procedure, participants were followed every 3-months for the first 2 years, and then every 6 months subsequently. Follow-up included clinical follow-up, PSA testing, administration of the American Urological Association Symptom Index (13) (AUA-SI) and the International Index of Erectile Function (14) (IIEF) questionnaires, as well as additional investigations as indicated. In addition to recording general complications during clinical follow-up, attempts were made to specifically assess and diagnose participants with HIFU specific complications including urethral strictures, osteitis pubis, and rectourethral fistulae. Routine post-HIFU biopsies were not performed.

The primary endpoint of this study was disease recurrence, defined as a composite endpoint of PSA progression by the ASTRO Phoenix criteria (10) (PSA nadir+2ng/ml), receipt of any further salvage therapy, receipt of ADT, clinical progression including the development of locally advancing disease or metastases, or death. Secondary analyses were performed to determine predictors of disease recurrence. Pre-specified predictors for which analysis was intended included TRUS biopsy grade, number of study entry TRUS biopsy cores positive, palpable disease at study enrollment, pre-HIFU PSA, an undetectable post-HIFU PSA nadir, and receipt of prior hormone therapy. An additional post-hoc analysis was added to determine if use of the Stuttgart criteria for biochemical failure after primary HIFU (PSA nadir+1.2ng/ml (15) would alter the number of participants classified as recurrence, with an intention to perform analyses utilizing both definitions of biochemical failure should this be the case.

Survival analysis with the Kaplan-Meier method was performed to determine disease recurrence-free survival (RFS) over time. Survival curves were stratified by each predictor of disease recurrence. A P value less than 0.05 on log-rank testing was used to determine whether the effect of each predictor on time to disease recurrence was statistically significant. Descriptive statistical analysis was performed with IBM SPSS Statistics version 22.0 (IBM Corporation, USA). Median values, range, and interquartile range (IQR) values are reported when appropriate.

## RESULTS

A total of 741 patients underwent HIFU at our center and 24 patients (3.2%) were eligible for study inclusion with a median follow-up of 31.0 months (range 12.3-70.2 months). Mean age at treatment was 68 years and 21/24 patients (88%) had received prior EBRT, while 3/24 (12%) had received prior brachytherapy. Most participants (21/24, 87.5%) had Gleason 7 or higher disease on study entry biopsy and 14/24 (58.3%) had palpable disease. Median PSA at study entry was 4.02ng/ml (range 0.90-28.90ng/ml). Patient demographics are listed in Table-1.

**Table 1:** Baseline characteristics of patients.

Variable	Value
Number of included patients (n)	24
Mean age (years)	68
Prior EBRT (n)	21 (88%)
Prior brachytherapy (n)	3 (12%)
Prior ADT (with radiotherapy) (n)	6 (25%)
Restaging MRI (n)	9 (38%)
Median PSA (ng/mL) [range]	4.02 [0.90 – 28.90] ng/mL
Gleason grade (n having 6, 7, 8-10)	3, 11, 10
Clinical T stage (n having T1, T2, T3)	10, 8, 6

**Figure 1:**
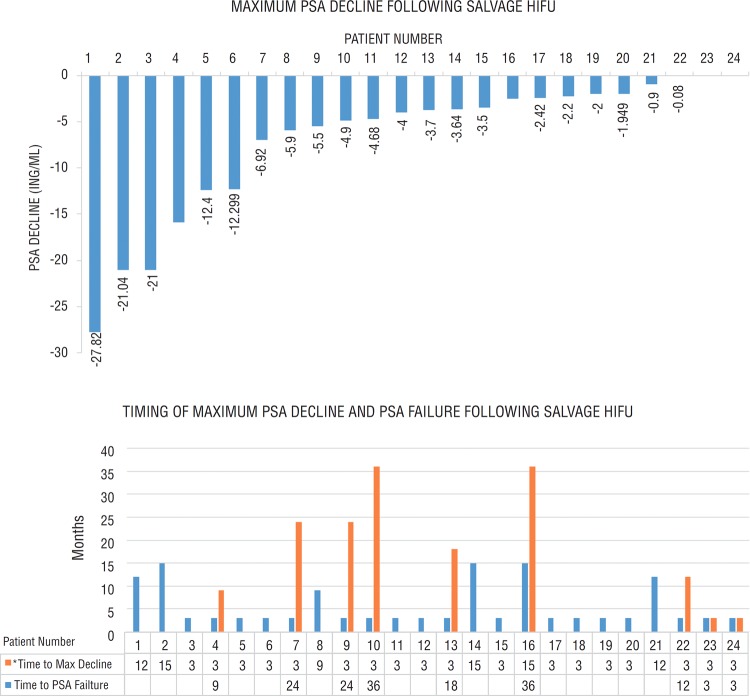
(A) Maximum PSA decline and (B) Timing of maximum PSA decline (post-HIFU nadir) and timing of PSA failure for individual patients treated with salvage HIFU.

All enrolled participants received salvage HIFU treatment as described in our protocol. Mean treated prostate volume was 23.8ml (range 11.3-38.8ml). No participants received ADT or transurethral resection of the prostate (TURP) as an adjunct to HIFU. Median time to PSA nadir was 3 months (range 3-15 months) after treatment and median post-HIFU PSA nadir was 0.04ng/ml (range 0-3.08). Two patients had no PSA responses post-HIFU (PSA increased at the 3-month post-HIFU measurement). Disease recurrence was experienced by 9 participants (38%) during follow-up with a median time to disease recurrence of 18 months (range 3-36 months). Disease recurrence was due to biochemical recurrence (rise of 2ng/ml above PSA nadir) in the 9 participants. If the alternate Stuttgart definition (PSA nadir+1.2ng/ml (14)) of biochemical failure was employed, none of the remaining 15 participants would be reclassified as treatment failures. Maximum PSA decline, timing of maximum PSA decline, and timing of biochemical recurrence is displayed for individual patients in Figure-1.

**Figure 2:**
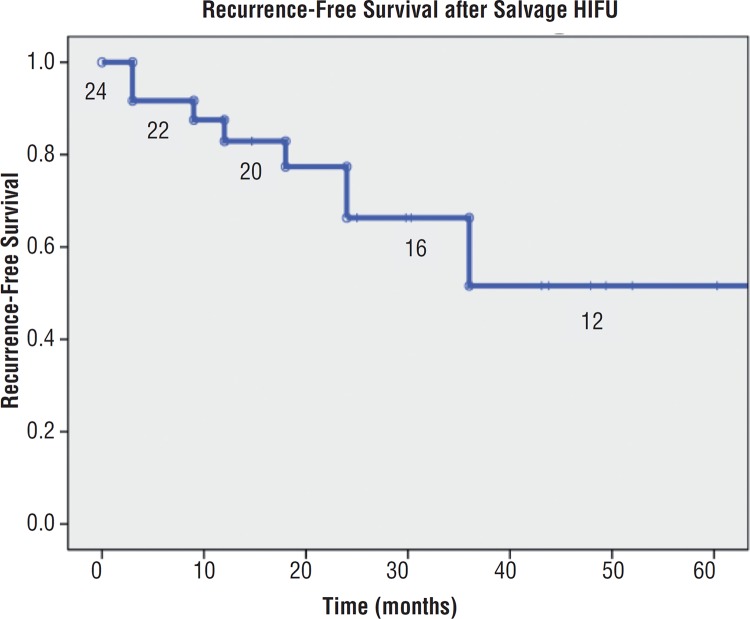
Recurrence-free survival (RFS) after salvage HIFU.

**Figure 3:**
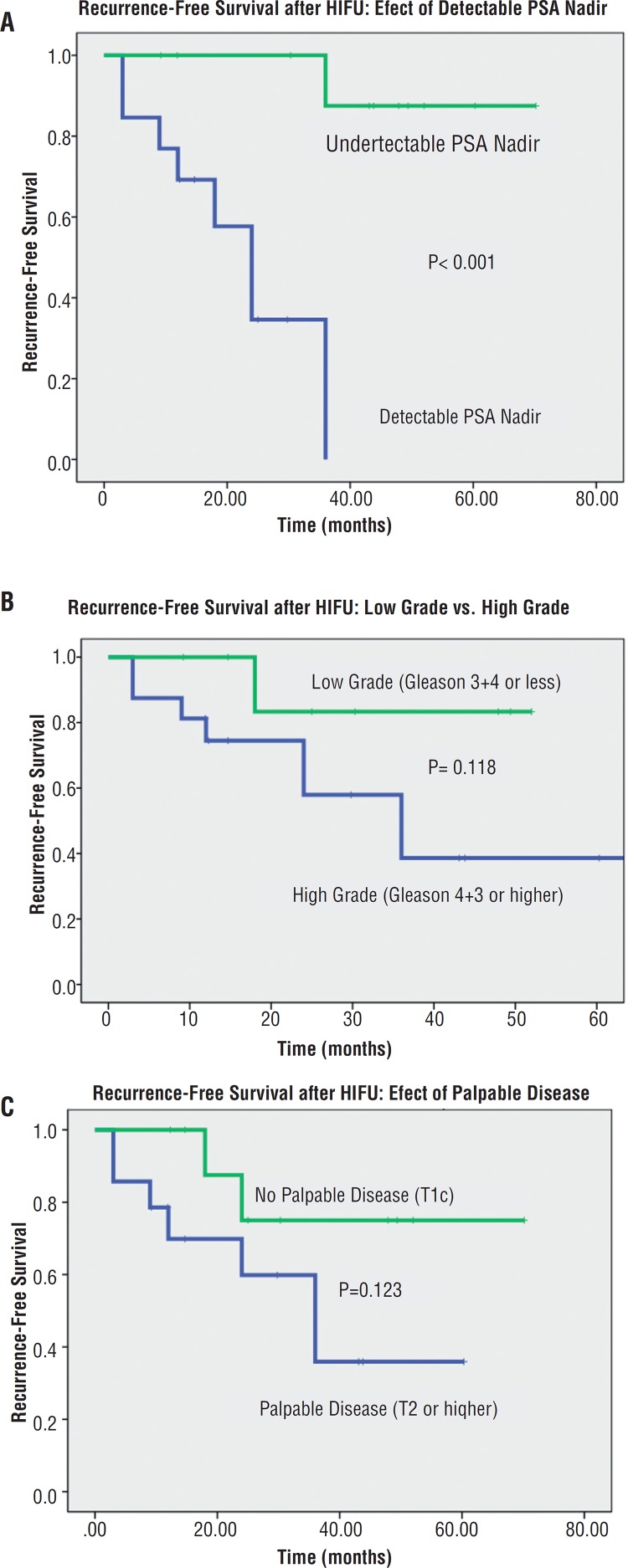
Subgroup analysis of recurrence-free survival (RFS) after salvage HIFU. (A) PSA nadir serves as an early predictor of RFS (P<0.001). (B) Low grade and (C) impalpable disease are suggestive of improved RFS but not statistically significant.

Overall survival analysis is displayed in Figure-2. Recurrence-free survival (RFS) after salvage HIFU was found to be 66.3% at 2-years and 51.6% at 5-years. Subgroup analyses of predictors of improved RFS shown in Figure-3 demonstrated that an undetectable PSA nadir predicted improved RFS (HR 0.07, 95% CI 0.02-0.29, log rank P<0.001). The remaining analyzed predictors, TRUS biopsy grade, number of study entry TRUS biopsy cores positive, palpable disease at study enrollment, pre--HIFU PSA, and receipt of prior hormone therapy, suggested an improved RFS but were not statistically significant (P>0.05). There was no difference in RFS between those patients initially treated with prior ERBT and brachytherapy.

One participant developed urethral stricture disease 9 months post-HIFU requiring visual internal urethrotomy (Clavien-Dindo Grade IIIb complication). No cases of rectourethral fistulae or osteitis pubis were observed. There were no other surgical complications noted. Median IPSS increased from a baseline of 8 (range 2-27, n=21) to 24 at a median year 1 average (range 6-32, n=16) and 17 at a median 2-year average (range 1-30, n=11). Median IIEF-15 declined from pre-treatment from 43 (n=17) to 19 post-treatment (n=12). IIEF data was limited in follow-up with most patients completing the post-treatment assessment at 6 months and not afterwards.

## DISCUSSION

With 20-60% of men receiving primary radiotherapy developing biochemical failure within 5 to 8 years, there is a clinical need to find curative salvage therapies (1, 2). Salvage prostatectomy may be potentially curative but carries a high risk of adverse events. Multiple studies have demonstrated cancer-specific survival to range from 70-83% and 54-89% at 5-years and 10-years respectively, and salvage prostatectomy has been reported to have 5-year and 10-year biochemical recurrence-free rates ranging from 47-82% and 28-53% respectively (5). Salvage prostatectomy is a more morbid operation than when performed primarily-previous series have demonstrated that salvage radical prostatectomy is associated with a 2-10% risk of rectal injury, 11-41% risk of anastomotic stricture, 80-100% risk of post-operative erectile dysfunction and 10-79% risk of incontinence.

The goal of an ablative therapy for prostate cancer is the maximum destruction of cancerous tissue with minimal injury to critical adjacent structures such as the urethra, urinary sphincter, bladder neck and rectum. In our study, we have found that approximately half of patients treated with salvage whole gland HIFU for radio-recurrent prostate cancer experienced intermediate-term RFS (2-year RFS: 66.3%, 5-year RFS: 51.6%). This is similar to other series that have reported intermediate term RFS ranging from 38-83% (Table-2).

**Table 2:** Review of literature for salvage HIFU (whole gland and focal) for radio-recurrent prostate cancer.

Study, Year	Device	No. patients (n)	Age (years)	Pre-HIFU PSA (months)	Median follow-up (months)	Primary therapy	Prior ADT (%)	Definition of biochemical failure	RFS	Incontinence (%)	Bladder obstruction (%)	Rectourethral fistula (%)	Osteitis Pubis (%)
Whole Gland HIFU													
Gelet, 2004	Ablatherm™	71	67	5.72	14.8	EBRT	30	ASTRO	38% (30 months)	35.2 (G3 = 7)	16.9	5.6	NR
Zacgarakis, 2008	Sonablate 500™	31	68	5.7	7.4	EBRT	58	ASTRO	71% (7.4 months)	6.5	36	6.5	NR
Murat, 2009	Ablatherm™	167	68	4.5	18.1	EBRT	56.8	Phoenix	53% low risk, 42% intermediate risk, 25% high risk (3 years)	49.5 (G3 = 9.5)	19.8	3	NR
Berge, 2010	Ablatherm™	46	67.4	5.5	9	EBRT	15.2	Phoenix	60.9% (9 months)	32.6	4.4	2.2	NR
						EBRT (63.6%),							
Uchida, 2010	Sonablate 500™	22	65	4	24	BT (22.7),	27.3	Phoenix	52% (5 years)	18.2	18.2	4.5	NR
						PT (13.6%)							
Ahmed, 2012	Sonablate 500™	84	68	4.3	19.8	EBRT	36	Phoenix	59% (1 year); 43% (2 year)	38	20	4.8	1.2
Crouzet, 2012	Ablatherm™	290	68.7	6.38	48	EBRT	50	Phoenix	45% low risk, 31% intermediate risk, 21% high risk (5 years)	46.6	16	2	2.7
Rouviere, 2013	Ablatherm™	46	NR	5.7	NR	EBRT	32.6	Phoenix	42% (2 year); 31% (4 year)	NR	NR	NR	NR
Song, 2014	Ablatherm™	13	68	4.63	44.5	ERBT	61.5	Stuttgart	53.8% (44.5 months)	30.8	38.5	0	NR
Yutkin, 2014	Sonablate 500™	19	66	3.25	59.3	BT	27	Stuttgart	66.7% (4.3 years)	31.6	21.1	15.8	0
Dason, 2016	Ablatherm™	24	68	4.02	31	ERBT (88%),	NR	Phoenix	66.3% (2 year);	NR	4.2	0	0
						BT (12%)			51.6% (5 year)				
Focal HIFU													
Ahmed, 2012	Sonablate 500™	39	70.5	3.3	17	EBRT	74.4	Phoenix	69% (1 year); 49% (2 year)	12.8	8	2.6	0
Baco, 2014	Ablatherm™	48	68.8	NR	16.3	EBRT (96%), BT (4%)	23	Phoenix	83% (1 year); 52% (2 year)	25	NR	0	2

In their retrospective analysis of 167 patients, Murat et al. reported local cancer control was achieved with negative biopsy results in 73% of patients and the 5-year overall survival rate was 84% (16). The 3-year progression-free survival rates were 53%, 42% and 25% for the low-, intermediate-, and high-risk groups, respectively. Murat et al. showed that those who had not had previous ADT, low pre-HIFU PSA and those with pre-radiotherapy low or intermediate D'Amico risk disease had improved RFS. Neither Gleason score nor positive biopsy percentage influenced RFS. Another study by Jones et al. of 100 patients at least 2 years after EBRT who received whole gland HIFU, 50 men achieved their 1 year endpoint of PSA nadir less than 0.5ng/mL and a negative biopsy (17).

A recent multicenter retrospective study by Crouzet et al. of 418 patients with radio--recurrent disease treated with HIFU showed that the overall survival, cancer specific survival and metastasis-free survival rates at 7 years were 72%, 82% and 81%, respectively (18). Pre-ERBT risk classification and pre-salvage HIFU PSA was shown to be associated with worse biochemical failure-free survival. Another study by Gelet et al. of 71 patients who were treated with salvage HIFU demonstrated that 80% had negative biopsies post HIFU and 61% had a nadir PSA obtained within 3 months of less than 0.5ng/mL (19). Mean follow--up was 14.8 months and at the last follow-up, 44% of patients had no evidence of any disease progression.

In our cohort, undetectable PSA nadir was the only identified predictor of improved RFS. This is validated by other data in the literature. Ahmed et al. showed that in 84 men who underwent whole gland salvage HIFU, PSA nadir >0.5ng/mL was predictive of failure (HR: 0.16, P<0.001) (2). The 1- and 2-year PFS for patients with a PSA nadir of <0.5ng/mL was 82 and 68% respectively, compared to 37 and 13% for those with a PSA nadir >0.5ng/mL. Neither pre-treatment PSA nor Gleason score were found to predict failure. Uchida et al. showed that in men with a PSA nadir <0.2ng/mL had a low rate of cancer detection on post--HIFU biopsy of only 11% compared to 54 and 52% if the PSA nadir was 0.21-1 or >1ng/mL respectively (20). This has led to some investigators suggesting that routine biopsy post-HIFU may not be required if the PSA nadir is less than 0.2ng/mL. The PSA nadir has also been shown to be a predictor of improved RFS in other setting including primary HIFU (21), focal salvage HIFU (22) and salvage cryoablation (23).

Other predictors of RFS shown by other investigators include pre-EBRT D'Amico risk (Murat), pre-HIFU PSA (8, 16, 21), previous ADT (8, 16), Gleason grade (8) and tumor extension anterior to the urethra as seen in MRI (21). However, these were not shown to be statistically significant in our cohort.

Compared to salvage prostatectomy, complication rates of salvage HIFU are hypothesized to be less severe (7). In our series, only 1 participant developed urethral stricture disease requiring visual internal urethrotomy and there were no cases of rectourethral fistulae or osteitis pubis. In a systematic review of HIFU for radio-recurrent prostate cancer, reported complications included a 2-16% risk of rectourethral fistula, 20% risk of bladder neck contracture, 10% risk of urethral strictures, 1.2-2.7% risk of osteitis pubis, 10-50% risk of incontinence and 66.2-100% risk of erectile dysfunction (24). Heterogeneity in adverse event reporting prevents a direct comparison of salvage HIFU toxicity to salvage prostatectomy.

Compared to whole gland salvage HIFU, focal HIFU is less well reported. Although preserving oncologic control while further minimizing harm may be achieved with focal therapy, difficulty with accurately localizing and treating recurrent disease may lead to progression and metastasis. Provisional data suggests similar oncological outcomes with lower adverse events compared to whole-gland ablation. Ahmed et al. reported on 39 patients treated with focal HIFU (hemi-ablation or focal ablation) for radio-recurrent prostate cancer (22). Progression-free survival rates at 1- and 2- years according to the Phoenix criteria were reported to be 69% and 49% respectively.

Baco et al. described 48 prospectively enrolled patients with radio-recurrent prostate cancer treated with hemi-salvage HIFU (25). Progression-free survival rates at 12, 18 and 24 months were 83%, 64% and 52% respectively. Disease progression occurred in 16 of 48 patients (33%). Of these, 4 had local recurrence in the untreated lobe, 4 had bilateral recurrence, 6 developed metastases and 2 had rising PSA without evidence of local recurrence or metastases. Thus, whole gland and focal salvage HIFU may provide a potential cure if patients are referred at an early stage when recurrence is suspected.

The present study's small sample size and short follow-up limited the confidence in estimation of RFS as well as the power of subgroup analyses and ability to analyze predictive factors. This study was also limited by a lack of follow-up biopsies to adequately demonstrate effective treatment. We defined treatment failure with a strict clinical definition. Further, purely PSA based definitions of biochemical failure including both the ASTRO definitions (10, 11) as well as the Stuttgart definition (15) are not validated to predict disease recurrence after salvage HIFU. This limits comparisons to salvage prostatectomy, for which a post--treatment PSA nadir of 0ng/ml is expected and biochemical recurrence is easily assessed.

Other than aggregate AUA-SI and IIEF-15 questionnaire data, adverse event recording was limited as we did not capture lower grade complications such as infections, hematuria, incontinence and perineal pain. Every attempt was made to assess for severe complications and we saw only 1 case of stricture disease requiring intervention (Clavien-Dindo Grade IIIb complication) and no cases of rectourethral fistulae or osteitis pubic. This is consistent with the low incidence of these complications as described in previous series (2, 8, 9). Additional complications may become evident with longer follow-up.

On routine follow-up, there was a median increase in IPSS at 1 year followed by subsequent improvement at 2-year follow-up. It has been previously reported that HIFU causes de novo detrusor overactivity and impaired bladder compliance seen on routine urodynamics in 10% of patients at 3 months, with progressive improvement at longer follow-up as seen in our series (26).

There is no consensus to date for the ideal candidate for salvage HIFU for radio-recurrent prostate cancer. The main candidate for salvage HIFU is a man that has radio-recurrent prostate cancer predicted to cause morbidity or mortality within his lifetime, but refuses or cannot receive salvage radical prostatectomy. Further, the effectiveness of salvage therapy is a function of how well metastases are excluded. We utilized standard bone scans and CT scans to rule out metastases. However, they are of limited value, especially when PSA is low. Some patients in this study may have had occult micrometastatasis at the time of HIFU and eventually had progression with distant disease leading to a failure of proper selection due to limitations of pre-HIFU screening or imaging. The patients who had no PSA response likely had unrecognized micrometastatic disease. Future studies on salvage therapy will rely on advanced imaging modalities to rule out systemic recurrence post curative treatment, including 18-F Choline PET and PSMA PET, which have reported sensitivities of 42-96% (27) and 66-89.5% (28).

In summary, salvage HIFU allows for inter-mediate-term disease control with acceptable morbidity in carefully selected patients with localized radio-recurrent prostate cancer. An undetectable PSA nadir, achieved at a median time of 3 months post-HIFU, serves as an early predictor of recurrence-free survival. Large multicenter trials with long-term follow-up are warranted to better assess oncological outcomes and adverse events.
